# The influencing factors of health-related quality of life among rural hypertensive individuals: a cross-sectional study

**DOI:** 10.1186/s12955-021-01879-6

**Published:** 2021-10-18

**Authors:** Shengxiang Sang, Ning Kang, Wei Liao, Xueyan Wu, Ze Hu, Xiaotian Liu, Chongjian Wang, Hongjian Zhang

**Affiliations:** 1 Department of Social Medicine and Health Management, College of Public Health, Zhengzhou University, Zhengzhou, Henan People’s Republic of China; 2 Department of Epidemiology and Biostatistics, College of Public Health, Zhengzhou University, 100 Kexue Avenue, Zhengzhou, 450001 Henan People’s Republic of China; 3 Department of Preventive Medicine, School of Medicine, Henan University of Chinese Medicine, Zhengzhou, Henan People’s Republic of China

**Keywords:** Health-related quality of life, Hypertension, EQ-5D-5L, Rural population, Socioeconomic status

## Abstract

**Background:**

Previous reports regarding health-related quality of life (HRQoL) of hypertensive individuals commonly concentrated on urban population. This study focused on rural population and aimed to explore the influencing factors of HRQoL.

**Methods:**

Date were derived from Henan Rural Cohort study. The HRQoL of participants were assessed via European Quality of Life Five Dimension Five Level Scale (EQ-5D-5L) instrument. Tobit regression model and generalized linear model were employed to explore the influencing factors of HRQoL. Another binary logistic regression was utilized to examine the robustness of our results.

**Results:**

Among 23,485 rural population, 8128 participants were identified with hypertension. The mean (SD) utility index and VAS score of non-hypertension group were 0.96 (0.09) and 79.66 (14.20), respectively, while in hypertension group were 0.94 (0.14) and 75.88 (15.50), respectively. Pain/discomfort was the most common self-reported problem (23.05%) for patients. Aging and suffering with other diseases were negatively associated with HRQoL among rural patients, while high socioeconomic status and healthy lifestyles corresponded with high HRQoL.

**Conclusions:**

Hypertension did push considerable pressures on patients’ HRQoL. Maintaining healthy lifestyles and improving the socioeconomic status of patients were advisable ways to reduce this burden.

*Trial registration* The Henan Rural Cohort Study has been registered at Chinese Clinical Trial Register (Registration number: ChiCTR-OOC-15006699). http://www.chictr.org.cn/showproj.aspx?proj=11375

**Supplementary Information:**

The online version contains supplementary material available at 10.1186/s12955-021-01879-6.

## Background

As the leading global risk factor for death and disability [1], hypertension has resulted in nearly 10.4 million deaths worldwide each year [2]. Despite of the rapid development of medical technology, the prevalence of hypertension was still disproportionate increasing [3]. Previous study indicated that in 2025, approximately two billion adults worldwide would be subjected to hypertension [4]. According to Global Burden of Disease Study, hypertension was identified as the second largest risk factor in China, causing nearly 2.1 million cardiovascular deaths and 1.2 million premature cardiovascular deaths each year [5]. Meanwhile, the disability-adjusted life years (DALYs) caused by hypertension in China was 37.94 million person-years and was 170 million person-years around the world, which demonstrated that hypertension did push considerable pressures on patients’ quality of life [6].

Regarded as a broad and multifaceted concept which usually reflected the physical and mental health status of individuals, health-related quality of life (HRQoL) was a widely mentioned in clinical and public health research [7]. In addition, a literature has reported HRQoL as an important intervention efficacy outcome for patients [8]. Various instruments have been proposed to precisely measure the HRQoL of individuals [9]. Composed a descriptive system including five dimensions and an EQ-Visual Analogue Scale system focusing on overall health status, European Quality of Life Five Dimension (EQ-5D) instrument was one of the most applicable measurements to assess HRQoL [10]. Compared with other counterparts, EQ-5D instrument was more applicable for people in rural areas with low education status and it could provide a quantitative measure of health outcome [11]. Additionally, the ceiling effect of EQ-5D instrument was reduced more with five level (EQ-5D-5L) than three level (EQ-5D-3L) [12].

Despite the diverse sources and divergent methods, sufficient researches had repeatedly indicated that the inverse association did exist between hypertension and HRQoL [13–15]. A cross-sectional study conducted in Hong Kong demonstrated that participants with hypertension had a low EQ-5D utility score, and high education status would alleviate this situation [16]. Considering that rural population had limited access to health care services accounted for lower control of hypertension [17], which may lead to less than optimal HRQoL [18], more attention should be paid in these specific areas. However, most previous researches focused on the negative association between HRQoL and hypertension rather than providing practical interventions to improve the HRQoL of hypertension participants. Thus, focusing on rural population, this research hypothesized that hypertension influenced HRQoL and further to explore the influencing factors on HRQoL of hypertensive patients.

## Methods

### Study participants

Subjects of this research came from Henan Rural Cohort study, a representative sample of rural population from Henan province in China. From July 2015 to September 2017, a total of 39,259 participants (response rate = 93.7%) aged from 18 to 79 years were enrolled from Henan rural areas via multistage stratified cluster sampling. In the first stage, five geographical regions (Yuzhou, Suiping, Tongxu, Xinxiang, and Yima) were selected through simple cluster sampling. In the second stage, several villages and towns were selected according to natural and medical conditions. In the end, all permanent residents aged 18–79 years and without severe physical or mental disease were enrolled as study sample. More details of this cohort were described in elsewhere [19].

In Henan Rural Cohort study, a total of 23,559 participants completed the EQ-5D-5L instrument (15,700 participants were excluded due to not invited). Then we further excluded 49 participants due to missing EQ-5D-5L information and 25 participants due to missing hypertension information. Finally, a total of 23,485 participants enrolled from Henan Rural Cohort were included in this analysis.

In full compliance with Declaration of Helsinki, ethical approval was received from the Zhengzhou University Life Science Ethics Committee (Code: [2015] MEC (S128)) and all procedures were conducted by trained staff. Considering the rationality of research, each participant was informed the purposes of survey and required to sign the consent form before the data collection. Both researchers and participants agreed to use this data for scientific research purposes only.

### Information sources and study variables

In Henan rural cohort, basic information of participants including demographic characteristics, lifestyle factors, and history of diseases were obtained from a standard questionnaire. Demographic characteristics consisted of age (18 ~ , 45 ~ , 65 ~), gender and socioeconomic status. Socioeconomic status included average monthly income of family (0 ~ , 500 ~ , 100 ~ , 2000 ~), education status (primary school or below, junior high school, and senior high school or above), and marital status (married/cohabiting, widowed/divorced/separated, and single). According to International Physical Activity Questionnaire (IPAQ), physical activity was assessed as low, moderate, and high level [20]. Sleep quality was evaluated via the Pittsburgh Sleep Quality Index (PSQI) and then categorized into two categories as poor (PSQI > 5) and good sleep quality (PSQI ≤ 5) [21].

Anthropometric measurements such as height and weight were measured by trained researchers. Height (nearest 0.1 cm) was measured with participants in an upright position against a calibrated wall and weight (nearest 0.1 kg) was measured with participants in light clothing and shoes off through standard measuring equipment. Body mass index (BMI) was calculated via dividing weight in kilograms by height in meters squared. Four other common non-communicable diseases (NCDs) including coronary heart disease (CHD), stroke, diabetes and dyslipidemia were assessed through physical examination, laboratory tests or self-reports.

### The measurement of blood pressure and definition of hypertension

Blood pressure was repeatedly measured three times by electronic sphygmomanometer (HEM-770AFuzzy, Omron, Japan). Before this measurement, each participant was asked to avoid caffeine, exercise, and smoking for no less than 30 min and had a 5 min rest in a seated position. The mean of the three measurements of each participant was used in the analysis. Hypertension was defined as mean systolic blood pressure (SBP) ≥ 140 mmHg or diastolic blood pressure (DBP) ≥ 90 mmHg, or a self-reported history of hypertension or had taken antihypertensive drugs in the last 2 weeks [22].

### Measurements of the HRQoL of participants

The HRQoL of participants in this research was assessed by EQ-5D-5L instrument, which consisted of two desperate parts as descriptive system and the EQ Visual Analogue scale (VAS) [23]. The information of descriptive system was collected with the assistance of interviewers, while the information of VAS was collected by self-completed. Descriptive system included five domains as mobility (MO), self-care (SC), usual activities (UA), pain/discomfort (PD) and anxiety/depression domain (AD) and each domain had five levels as no problems, slight problems, moderate problems, severe problems and extreme problems. The utility index of descriptive system was calculated by the latest available Chinese value set [24].

Utility index ranged from − 0.391 to 1.000, in which 1.000 represented the full health status. Any problem in five domains was classified as impaired health status [25]. Recording the self-rated health of respondents, EQ VAS was a continuous variable ranged from 0 (the worst imaginable state of health) to 100 (the best imaginable state of health).

### Statistical analysis

Characteristics of continuous variables were shown as means with standard deviations (SD) and the intergroup variances were detected by Student’s t-tests, whereas categorical variables were expressed by frequency with percentage and the intergroup differences were compared through chi-squared tests. After adjustment of covariates, logistic regression model was employed to detected the association between hypertension and HRQoL. Among hypertensive participants, the potential influencing factors of VAS score was detected via generalized linear model (GLM). Considering the extremely skewed distribution (Additional file 1: Fig. S1), a tobit regression model was employed in utility index [26]. The influencing factors of each domain in descriptive system were detected by logistic regression. To assess the robustness of the results, participants were divided into health group (utility index = 1) and unhealth group (utility index < 1) [25], and the potential risking factors were detected by logistic regression model. Considering the data is large enough, a cross-validation methodology was also adopted. Moreover, *Cohen’s D* was utilized to estimate the effect size of hypertension on HRQoL [27]. When the *Cohen’s D* greater than or equal to 0.15 and less than 0.40, it is classified as small; when it is greater than or equal to 0.40 and less than 0.70, it is classified as medium; when it is greater than or equal to 0.70, it is classified as large. The medium effect size was considered clinically significant.

Analyses in this research were conducted via Statistical Package for the Social Sciences version 21.0 (IBM-SPSS Inc, Armonk, NY) and R version 4.0.3. Findings at P < 0.05 were regarded as significant.

## Results

### Characteristics of study participants

A total of 9533 men and 13,952 women were finally enrolled in this research, including 8128 (34.61%) hypertensive participants and 15,357 (65.39%) non-hypertensive participants. The mean age of hypertension group was 60.52 (10.25) years old, whereas in non-hypertension group was 52.52 (12.88). Compared with non-hypertensive participants, participants with hypertension had following characteristics: men, lower education status, more proportion of unmarried/divorced/widowed, lower family average monthly income, lower physical activity, poorer sleep quality, and more probability to suffer from other NCDs. Regarding the HRQoL of participants, both in utility index and VAS score, non-hypertension group (0.96 ± 0.09, 79.66 ± 14.20) had a significantly higher score than hypertension group (0.94 ± 0.14, 75.88 ± 15.50). More baseline characteristics are detailed in Table 1. After adjustment of covariates, results indicated that hypertension did have a significantly negative association with HRQoL, although this association was not clinically significant (Additional file 1: Table S1).

**Table 1:** Summary statistics of the characteristics for all participants

Variable	Total	Hypertension	Non-hypertension	*P*
	(N = 23,485)	(N = 8128)	(N = 15,357)	
Age (year, mean ± SD)	55.29 ± 12.62	60.52 ± 10.25	52.52 ± 12.88	< 0.001
*Gender (n, %)*				0.017
Men	9533 (40.59)	3385 (41.65)	6148 (40.03)	
Women	13,952 (59.41)	4743 (58.35)	9209 (59.97)	
*Education status (n, %)*				< 0.001
Primary or below	10,108 (43.04)	4253 (52.33)	5855 (38.13)	
Middle school	8971 (38.20)	2695 (33.16)	6276 (40.87)	
High school or above	4406 (18.76)	1180 (14.52)	3226 (21.01)	
*Marital status (n, %)*				< 0.001
Married/cohabiting	21,185 (90.21)	7149 (87.96)	14,036 (91.40)	
Widowed/divorced/separated/single	2300 (9.79)	979 (12.04)	1321 (8.60)	
*Per capita monthly income (RMB, n, %)*				< 0.001
< 500	8679 (36.96)	3311 (40.74)	5368 (34.95)	
500 ~	7444 (31.70)	2629 (32.34)	4815 (31.35)	
1000 ~	5348 (22.77)	1667 (20.51)	3681 (23.97)	
2000 ~	2014 (8.58)	521 (6.41)	1493 (9.72)	
*Physical activity (n, %)*				< 0.001
Low	8113 (34.55)	3189 (39.23)	4924 (32.06)	
Moderate	7814 (33.27)	2393 (29.44)	5421 (35.30)	
High	7558 (32.18)	2546 (31.32)	5012 (32.64)	
*Sleep quality (n, %)*				< 0.001
Good	18,501 (79.39)	6190 (76.89)	12,311 (80.71)	
Poor	4802 (20.61)	1860 (23.11)	2942 (19.29)	
*NCDs (n, %)*				< 0.001
Non	11,889 (50.75)	2967 (36.61)	8922 (58.22)	
One	8821 (37.65)	3540 (43.68)	5281 (34.46)	
Two	2314 (9.88)	1320 (16.29)	994 (6.49)	
Three or more	404 (1.72)	277 (3.42)	127 (0.83)	
BMI (kg/m^2^, mean ± SD)	24.98 ± 3.59	26.19 ± 13.32	24.35 ± 11.52	< 0.001
Utility index (mean ± SD)	0.95 ± 0.11	0.94 ± 0.14	0.96 ± 0.09	< 0.001
VAS score (mean ± SD)	78.33 ± 14.80	75.88 ± 15.50	79.66 ± 14.20	< 0.001

T-test was performed to compare the differences in continuous variables; Chi-square test was used to compare the differences in the categorical variables

SD, standard deviation; RMB, Renminbi; NCDs, non-communicable diseases

### Self-reported HRQoL in five domains

**Fig. 1:**
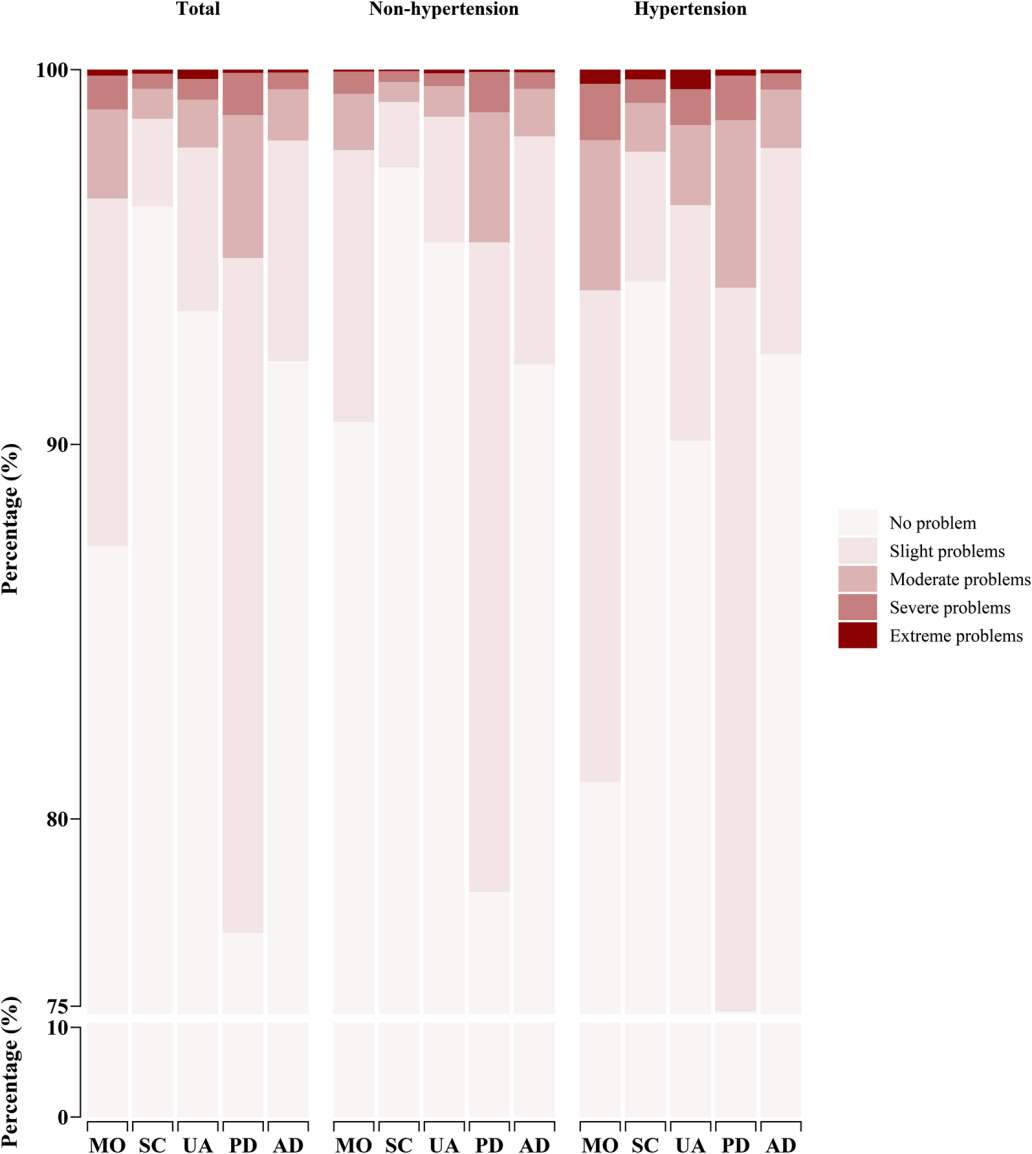
Distribution of the EQ-5D-5L by self-classified health states

Figure 1 summarizes the self-reported HRQoL of participants based on the descriptive system. Followed by mobility domain (19.01%), pain/discomfort domain (23.05%) was the most common self-reported problem in hypertension group, as well as in non-hypertension group (9.40%, 21.95%). Changed from 9.40 to 19.10%, mobility (19.01% versus 9.40%) domain was the most vulnerable domain for patients. Although self-care domain was the least self-reported problem, the difference between hypertension group (5.66%) and non-hypertension (2.60%) was still significant. Additionally, significant variance between hypertension and non-hypertension group was also found (both *P* < 0.001) usual activity (9.90% versus 4.61%) and pain/discomfort (23.05% versus 21.95%) domain. However, the variance in anxiety/depression domain was not detected (*P* = 0.133). Detailed information is presented in Additional file 1: Table S2.

### Influencing factors of HRQoL of hypertensive patients

**Fig. 2:**
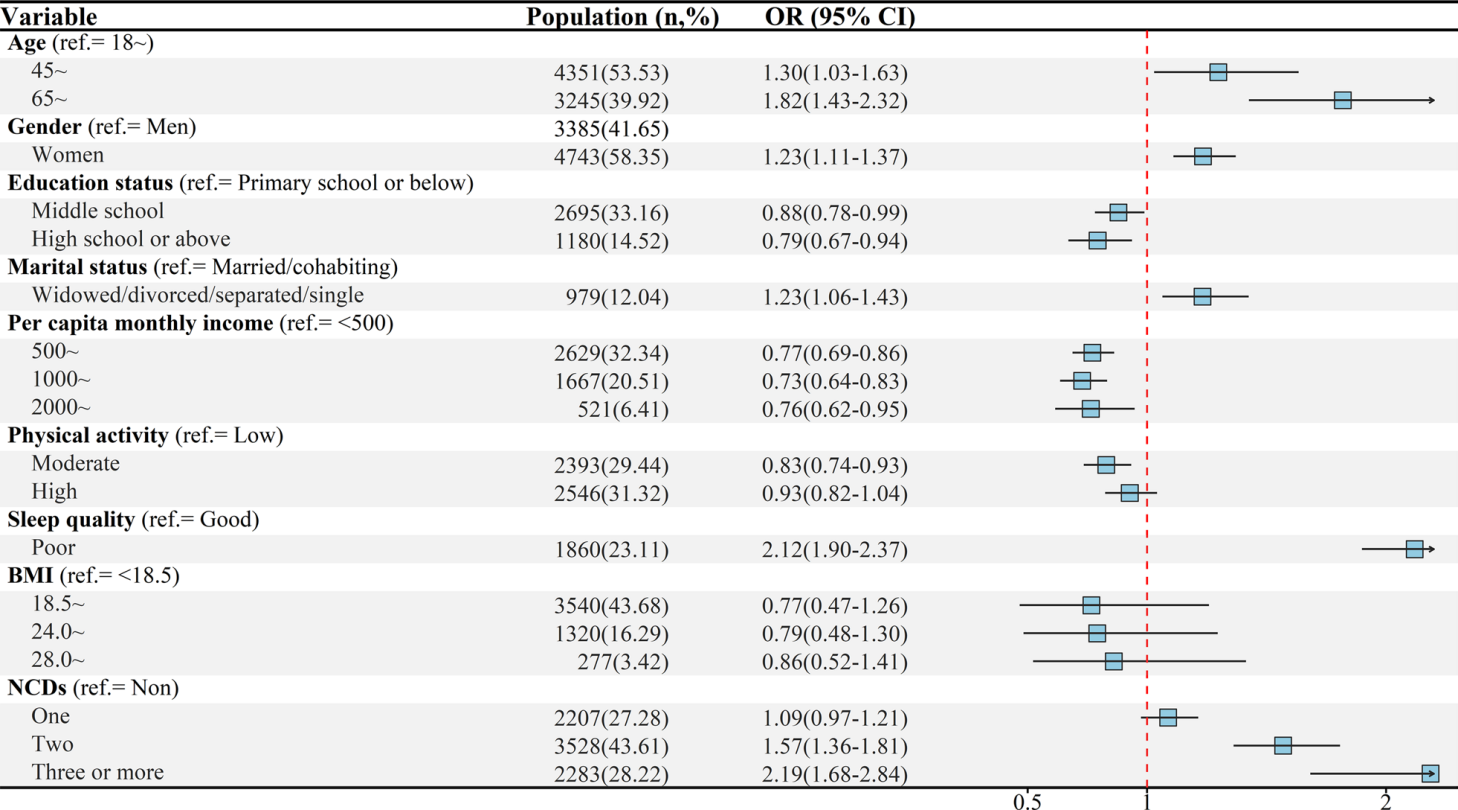
The results of sensitive analyses

The results of tobit regression on utility index and generalized linear regression on VAS score are displayed in Table 2. Both in utility index and VAS score, aging (beta = − 0.10, − 4.96, respectively), poor sleep quality (− 0.12, − 6.44), and suffering from other NCDs (− 0.18, − 12.97) were negatively associated with HRQoL of hypertensive patients, while higher education status (0.05, 1.70), higher monthly income (0.07, 3.42), and high physical activity (0.04, 2.15) were positively associated with HRQoL. An inverse association was observed between HRQoL and women (− 0.02) as well as poor marital status (− 0.02) in utility index, while the significant association was not detected in VAS score. The significant association between HRQoL and BMI was only found in VAS score. Presented in Fig. 2, the results of sensitive analyses show a similar consequence.

**Table 2:** Multivariable linear regression model on utility index and VAS score among patients

Variable	Utility index			VAS score		
	*Coe*	*SE*	*P*	*Coe*	*SE*	*P*
*Gender (ref.* = *Men)*						
Women	− 0.02	0.08	0.004	0.22	0.20	0.271
*Age (ref.* = *18* ~*)*						
45 ~	− 0.04	0.02	0.014	− 2.79	0.26	< 0.001
65 ~	− 0.10	0.02	< 0.001	− 4.96	0.32	< 0.001
*Education status (ref.* = *Primary school or below)*						
Middle school	0.03	0.01	0.001	0.68	0.22	0.002
High school or above	0.05	0.01	< 0.001	1.70	0.29	< 0.001
*Marital status (ref.* = *Married/cohabiting)*						
Widowed/divorced/separated/single	− 0.02	0.01	0.04	0.09	0.32	0.771
*Per capita monthly income (ref.* < *500)*						
500 ~	0.05	0.01	< 0.001	2.09	0.22	< 0.001
1000 ~	0.06	0.01	< 0.001	2.97	0.25	< 0.001
2000 ~	0.07	0.02	< 0.001	3.42	0.36	< 0.001
*Physical activity (ref.* = *Low)*						
Moderate	0.05	0.01	< 0.001	1.62	0.22	< 0.001
High	0.04	0.01	< 0.001	2.15	0.23	< 0.001
*Sleep quality (ref.* = *Good)*						
Poor	− 0.12	0.01	< 0.001	− 6.44	0.23	< 0.001
*BMI (kg/m*^*2*^*) (ref.* = < *18.5)*						
18.5 ~	0.02	0.04	0.504	2.45	0.63	< 0.001
24.0 ~	0.02	0.04	0.555	3.04	0.64	< 0.001
28.0 ~	0.01	0.04	0.818	2.92	0.66	< 0.001
*NCDs (ref.* = *Non)*						
One	− 0.01	0.01	0.088	− 1.16	0.20	< 0.001
Two	− 0.08	0.01	< 0.001	− 5.40	0.33	< 0.001
Three or more	− 0.18	0.02	< 0.001	− 12.97	0.72	< 0.001

Tobit regression model was utilized to estimate the potential influencing factors of Utility index

Generalized linear model was utilized to assess the potential influencing factors of VAS-score

Table 3 describes the influencing factors on HRQoL of patients in five separated domains. Compared with men, women were negatively associated with HRQoL in pain/discomfort (OR 1.27 (1.13–1.43)) and anxiety/depression (OR 1.29 (1.07–1.57)) domains. An inverse association was also detected between aging and HRQoL in all domains with the exception of anxiety/depression domain (OR 0.62 (0.42–0.92)). Regarding to socioeconomic status, although higher monthly income corresponded to higher HRQoL in all five domains (all *P* < 0.05), the same association was not discerned between education status and HRQoL in anxiety/depression domain. The association between marital status and HRQoL was plausible and was only slightly observed in pain/discomfort (OR 1.26 (1.08–1.47)) and anxiety/depression (OR 1.35 (1.06–1.73)) domains. Additionally, poor sleep quality and suffering with other NCDs were negatively associated with HRQoL in all domains.

**Table 3:** The influencing factors on five domains among hypertensive individuals

Variable	Mobility	Self-care	Usual activities	Pain/discomfort	Anxiety/depression
*Gender (ref.* = *Men)*					
Women	1.06 (0.93–1.21)	0.83 (0.67–1.04)	**0.84 (0.71–1.00)**	**1.27 (1.13–1.43)**	**1.29 (1.07–1.57)**
*Age (ref.* = *18* ~*)*					
45–64	**3.65 (2.22–6.00)**	2.26 (0.91–5.60)	**2.18 (1.20–3.96)**	1.29 (0.99–1.68)	**0.66 (0.46–0.95)**
65–79	**6.98 (4.22–11.55)**	**4.04 (1.62–10.09)**	**3.73 (2.04–6.81)**	**1.76 (1.33–2.32)**	**0.62 (0.42–0.92)**
*Education status (ref.* = *Primary school or below)*					
Middle school	**0.82 (0.71–0.95)**	**0.66 (0.51–0.86)**	**0.66 (0.54–0.80)**	**0.84 (0.74–0.96)**	1.08 (0.88–1.33)
High school or above	**0.72 (0.58–0.91)**	**0.48 (0.31–0.76)**	**0.63 (0.46–0.86)**	**0.73 (0.60–0.88)**	1.16 (0.87–1.55)
*Marital status (ref.* = *Married/cohabiting)*					
Widowed/divorced/separated/single	1.14 (0.97–1.35)	1.18 (0.91–1.54)	1.16 (0.94–1.44)	**1.26 (1.08–1.47)**	**1.35 (1.06–1.73)**
*Per capita monthly income (ref.* < *500)*					
500 ~	**0.68 (0.60–0.79)**	**0.67 (0.53–0.85)**	**0.70 (0.58–0.83)**	**0.78 (0.69–0.88)**	**0.75 (0.61–0.92)**
1000 ~	**0.59 (0.50–0.70)**	**0.57 (0.42–0.78)**	**0.62 (0.49–0.77)**	**0.74 (0.64–0.86)**	0.85 (0.67–1.07)
2000 ~	**0.51 (0.38–0.70)**	**0.34 (0.17–0.67)**	**0.41 (0.26–0.65)**	0.83 (0.65–1.05)	0.69 (0.46–1.03)
*Physical activity (ref.* = *Low)*					
Moderate	**0.62 (0.53–0.72)**	**0.65 (0.51–0.83)**	**0.57 (0.47–0.69)**	0.94 (0.82–1.07)	0.94 (0.76–1.16)
High	**0.62 (0.54–0.72)**	**0.43 (0.33–0.56)**	**0.49 (0.40–0.59)**	1.09 (0.96–1.24)	0.95 (0.77–1.17)
*Sleep quality (ref.* = *Good)*					
Poor	**1.92 (1.69–2.19)**	**2.64 (2.15–3.24)**	**2.20 (1.87–2.59)**	**1.99 (1.77–2.23)**	**2.58 (2.16–3.07)**
*BMI (kg/m*^*2*^*) (ref.* < *18.5)*					
18.5 ~	0.73 (0.41–1.29)	1.13 (0.43–2.95)	1.66 (0.69–3.98)	1.03 (0.60–1.79)	0.54 (0.27–1.09)
24.0 ~	0.88 (0.50–1.54)	0.99 (0.38–2.59)	1.56 (0.65–3.74)	1.09 (0.63–1.89)	**0.43 (0.21–0.87)**
28.0 ~	1.05 (0.60–1.86)	1.18 (0.45–3.11)	1.90 (0.79–4.58)	1.18 (0.68–2.05)	**0.44 (0.21–0.89)**
*NCDs (ref.* = *Non)*					
One	1.07 (0.93–1.24)	1.22 (0.94–1.58)	1.19 (0.98–1.44)	1.04 (0.92–1.18)	1.12 (0.92–1.38)
Two	**1.52 (1.28–1.80)**	**2.18 (1.64–2.88)**	**1.82 (1.46–2.27)**	**1.47 (1.26–1.71)**	**1.87 (1.47–2.38)**
Three or more	**2.32 (1.75–3.07)**	**3.31 (2.22–4.94)**	**3.04 (2.20–4.21)**	**1.65 (1.26–2.16)**	**2.68 (1.85–3.88)**

Bold fonts were significant odds ratios

## Discussion

Located in central China, Henan province is a typical agricultural, population, and moderately developed province, in which 79% population were in rural areas. Most population in Henan rural areas engage in agricultural production and others also work in small factories. Additionally, the dramatic urbanization is taking place in Henan province. Based on this large rural population, this research provided up-to-data evidence on HRQoL of hypertensive patients and its influencing factors in resource limited areas. Compared with the health group, hypertension group had a worse utility index and VAS score, which indicated that an inverse association did exist between hypertension and HRQoL. The most vulnerable domain for hypertensive patients was pain/discomfort while higher BMI may alleviate the discomfort in anxiety/depression domain. Regarding to the influencing factors, a negative association was identified between HRQoL and women as well as aging, poor sleep quality and suffering with other diseases, while positive association was detected between HRQoL and well socioeconomic status (high education status or high monthly income) and adequate physical activities.

Rapid economic expansion in urban areas led to large migration from rural to urban areas and non-labor population was left, which may account for a higher average age (55.29 ± 12.62) in this research [28]. Although conducted in different areas and assessed via disparate instruments, an impaired HRQoL of hypertensive patients was detected in our research as well [29]. Previous research regarding HRQoL with EQ-5D in Chinese old rural population also demonstrated that hypertension did contribute to HRQoL loss [30]. Compared with 0.87 in a similar research conducted in 2013 [31], the utility index of hypertensive patients was slightly higher in this research (0.94 ± 0.14), and the different participants as well as the improved life condition in rural areas may explain this variance [32]. With the further improvement of medical security system such as the popularization of rural cooperative medical care system [33], the costs for hypertension have decreased, which remarkably alleviated the anxiety and depression disorders for most patients. Thus, the significant difference between health people and patients in anxiety/depression domain was not detected. Similar to the previous study, self-care was the least frequently reported problem [18]. Considering the positive association between hypertension and chronic pain has been distinguished long time ago [34], it was reasonable to found that pain/discomfort domain was the most common self-reported problem for hypertensive patients. Previous systematic review included 20 Chinese publications also indicated that pain/discomfort domain was the most problems reported by patients and suffering from other chronic diseases also accounted for HRQoL loss [35].

Aging has been reported to be positively associated with hypertension [36]. In this research, not only did we find a higher age in hypertension group, but we also found a lower HRQoL was associated with increasing age for patients. Previous study conducted in Shananxi also reported that utility index was decreasing with age, also this trend was not significant [31]. A previous study has indicated that the complications including vascular, microvascular, and macrovascular increased with aging for hypertensive individuals [37], which corresponded to a lower HRQoL in old patients. Gender was also a crucial factor in HRQoL of hypertensive individuals. Similar study conducted in Jiangsu also reported that women was negative associated with HRQoL among patients [18]. Compared with men, women were more likely to neglect their hypertension symptoms [38]. This limited treatment hypertension condition would make women more vulnerable to complications, which hinted that an inverse association between women and HRQoL was reasonable [39]. Besides above unmodified factors, our research also observed a positive association between socioeconomic status and HRQoL among patients, which means higher monthly income or higher education status would alleviate the adverse effects of hypertension. Although socioeconomic status was not directly related to disease, higher monthly income affected the quality of health service and higher educational level was associated with more awareness of diseases, which directly affected the HRQoL [40]. Additionally, previous study indicated that participants with low socioeconomic may be more vulnerable to the effects of unhealthy lifestyle factors [41]. Thus, it was of great significance to improve the educational amenities of rural areas. In line with previous consensus, our research also found that adequate physical activities would improve the HRQoL [42]. Due to the impact of BMI on HRQoL varied by gender, the significant association among patients was not detected in our research [43]. However, a plausible association was observed in anxiety/depression domain, which indicted that patients with high BMI would maintain a better mental status. Previous researches among rural population repeatedly demonstrated that obesity was negatively associated with HRQoL [30, 35], but another study observed that high BMI may have a better mental status than low BMI participants [44]. The different classification of BMI may account for this difference and a previous study use a same classification of BMI with this research had a similar result [18]. The positive association between sleep quality and HRQoL was also detected in our results. Considering short sleep or poor sleep quality would increase the incidence of hypertension and other comorbidities, a good sleep quality should be advocated for all patients [45].

This study presented an up-to-data complement to influencing factors on HRQoL of hypertensive individuals in rural areas. Compared with other researches, this study has various advantages. Firstly, large sample of population and well-trained staff with standardized tools made the results more authentic and convincing. Secondly, analyses in five divergent domains would distinguish more influencing factors on HRQoL. Additionally, the sensitive analysis made the research more rigorous. However, some limitations should be noted. Firstly, this study was a cross-sectional study, so it was impossible to determine the causal relationship between HRQoL and influencing factors. Secondly, four common diseases were integrated in NCDs and there was no detailed description of a single disease, which may neglect the diverse effects of different diseases. Moreover, due to only rural population were included in this research, generalizing our founding to general population was restricted.

## Conclusion

Evidences in this study demonstrated hypertension was inversely associated with HRQoL among rural population and patients with less education status, lower per capita monthly income, less physical activity and poorer sleep quality, and suffering with other NCDs had lower HRQoL. Thus, to improve the HRQoL and reduce the burden of hypertension, it is important to increase the wages of patients, which increasing the investment in rural basic education and vocational education may be an effective way. In a long run, improving the neighborhood availability of sport facilities and appealing for a healthy lifestyle are still essential. Moreover, the fully-covered social security institutions should be strengthen and targeted policies to old population regarding medical services should be put forward.

## Supplementary Information

**Additional file 1**.** Supplementary appendix: Supplementary table 1**. The association between hypertension and HRQoL.** Supplementary table 2**. Self-reported health problems of respondents.** Supplementary table 3**. Results of 10-folds cross-validation.** Supplementary figure 1**. The distribution of utility index of hypertensive patients.

## Data Availability

Not applicable.

## Abbreviations

HRQoL: Health related quality of life
EQ-5D-5L: European Quality of Life Five Dimension Five Level Scale
SD: Standard deviation
DALYs: Disability-adjusted life years
BMI: Body mass index
SBP: Systolic blood pressure
DBP: Diastolic blood pressure
NCDs: Non-communicable diseases
CHD: Coronary heart disease
VAS: Visual Analogue scale
MO: Mobility
SC: Self-care
UA: Usual activities
PD: Pain/discomfort
AD: Anxiety/depression
OR: Odds ratio
CI: Confidence interval
