# Supplementary material for: The influencing factors of health-related quality of life among rural hypertensive individuals: a cross-sectional study
Source: Health Qual Life Outcomes. 2021 Oct 18;19:244. doi: 10.1186/s12955-021-01879-6 (PMC8524889; doi:10.1186/s12955-021-01879-6)

**Supplementary appendix**

This appendix formed part of the original submission and has been peer reviewed. We post it as supplied by the authors.

**Supplement to:** The influencing factors of health-related quality of life among rural hypertensive individuals: a cross-sectional study

**Table Legends**

**Supplementary table 1.** The association between hypertension and HRQoL.

**Supplementary table 2.** Self-reported health problems of respondents.

**Supplementary table 3.** Results of 10-folds cross-validation.

**Figure Legends**

**Supplementary figure 1.** The distribution of utility index of hypertensive patients.

**Supplementary table 1. The association between hypertension and HRQoL.**

| Variable | Utility index | | | |  | VAS score | | | |
| --- | --- | --- | --- | --- | --- | --- | --- | --- | --- |
|  | *Coe* | *SE* | *P* | *Cohen’s D* |  | *Coe* | *SE* | *P* | *Cohen’s D* |
| HTN (ref.=No) |  |  |  |  |  |  |  |  |  |
| Yes | -0.010 | 0.004 | 0.019 | 0.222 |  | -1.483 | 0.208 | <0.001 | 0.266 |

1. Adjusted by age, gender, education level, marry status, per capita monthly income, physical activity, sleep quality, NCDs, and BMI.
2. When the *Cohen’s D* greater than or equal to 0.15 and less than 0.40, it is classified as small; when it is greater than or equal to 0.40 and less than 0.70, it is classified as medium; when it is greater than or equal to 0.70, it is classified as large.

**Supplementary table 2. Self-reported health problems of respondents.**

| Variable | Total | Hypertension | Non-hypertension | *P* |
| --- | --- | --- | --- | --- |
|  | (N=23485) | (N=8128) | (N=15357) |  |
| Mobility (n, %) |  |  |  | <0.001 |
| No problems | 20497(87.28) | 6583(80.99) | 13914(90.60) |  |
| Slight problems | 2180(9.28) | 1066(13.12) | 1114(7.25) |  |
| Moderate problems | 558(2.38) | 326(4.01) | 232(1.51) |  |
| Sever problems | 212(0.90) | 122(1.50) | 90(0.59) |  |
| Extreme problems | 38(0.16) | 31(0.38) | 7(0.05) |  |
| Self-care (n, %) |  |  |  | <0.001 |
| No problems | 22625(96.34) | 7668(94.34) | 14957(97.40) |  |
| Slight problems | 550(2.34) | 282(3.47) | 268(1.75) |  |
| Moderate problems | 188(0.80) | 106(1.30) | 82(0.53) |  |
| Sever problems | 95(0.40) | 51(0.63) | 44(0.29) |  |
| Extreme problems | 27(0.11) | 21(0.26) | 6(0.04) |  |
| Usual activity (n, %) |  |  |  | <0.001 |
| No problems | 21972(93.56) | 7323(90.10) | 14649(95.39) |  |
| Slight problems | 1025(4.36) | 511(6.29) | 514(3.35) |  |
| Moderate problems | 300(1.28) | 174(2.14) | 126(0.82) |  |
| Sever problems | 130(0.55) | 78(0.96) | 52(0.34) |  |
| Extreme problems | 58(0.25) | 42(0.52) | 16(0.10) |  |
| Pain/discomfort (n, %) | | | | <0.001 |
| No problems | 18071(76.95) | 6085(74.86) | 11986(78.05) |  |
| Slight problems | 4233(18.02) | 1570(19.32) | 2663(17.34) |  |
| Moderate problems | 896(3.82) | 363(4.47) | 533(3.47) |  |
| Sever problems | 263(1.12) | 97(1.19) | 166(1.08) |  |
| Extreme problems | 22(0.09) | 13(0.16) | 9(0.06) |  |
| Anxiety/depression (n, %) | | | | 0.133 |
| No problems | 21658(92.22) | 7511(92.41) | 14147(92.12) |  |
| Slight problems | 1383(5.89) | 447(5.50) | 936(6.09) |  |
| Moderate problems | 322(1.37) | 127(1.56) | 195(1.27) |  |
| Sever problems | 103(0.44) | 35(0.43) | 68(0.44) |  |
| Extreme problems | 19(0.08) | 8(0.10) | 11(0.07) |  |

**Supplementary table 3. Results of 10-folds cross-validation.**

| **Variable** | **Utility index** |  | **VAS score** |
| --- | --- | --- | --- |
|  | *Coe* |  | *Coe* |
| Gender (ref. = Men) |  |  |  |
| Women | -0.02 |  | 0.23 |
| Age (ref. = 18~) |  |  |  |
| 45~ | -0.04 |  | -1.77 |
| 65~ | -0.10 |  | -3.71 |
| Education status (ref. = Primary school or below) | |  |  |
| Middle school | 0.03 |  | 0.26 |
| High school or above | 0.04 |  | 1.36 |
| Marry status (ref. = Married/cohabiting) | |  |  |
| Widowed/divorced/separated/single | -0.02 |  | -0.05 |
| Per capita monthly income (ref. <500) | |  |  |
| 500~ | 0.05 |  | 2.34 |
| 1000~ | 0.06 |  | 3.81 |
| 2000~ | 0.06 |  | 5.17 |
| Physical activity (ref. = Low) |  |  |  |
| Moderate | 0.05 |  | 1.85 |
| High | 0.04 |  | 2.90 |
| Sleep quality (ref. = Good) |  |  |  |
| Poor | -0.12 |  | -5.97 |
| BMI (kg/m^2^) (ref. = <18.5) |  |  |  |
| 18.5~ | 0.03 |  | 2.28 |
| 24.0~ | 0.03 |  | 1.74 |
| 28.0~ | 0.02 |  | 2.78 |
| NCDs (ref. = Non) |  |  |  |
| One | -0.02 |  | -1.47 |
| Two | -0.08 |  | -5.94 |
| Three or more | -0.19 |  | -14.40 |


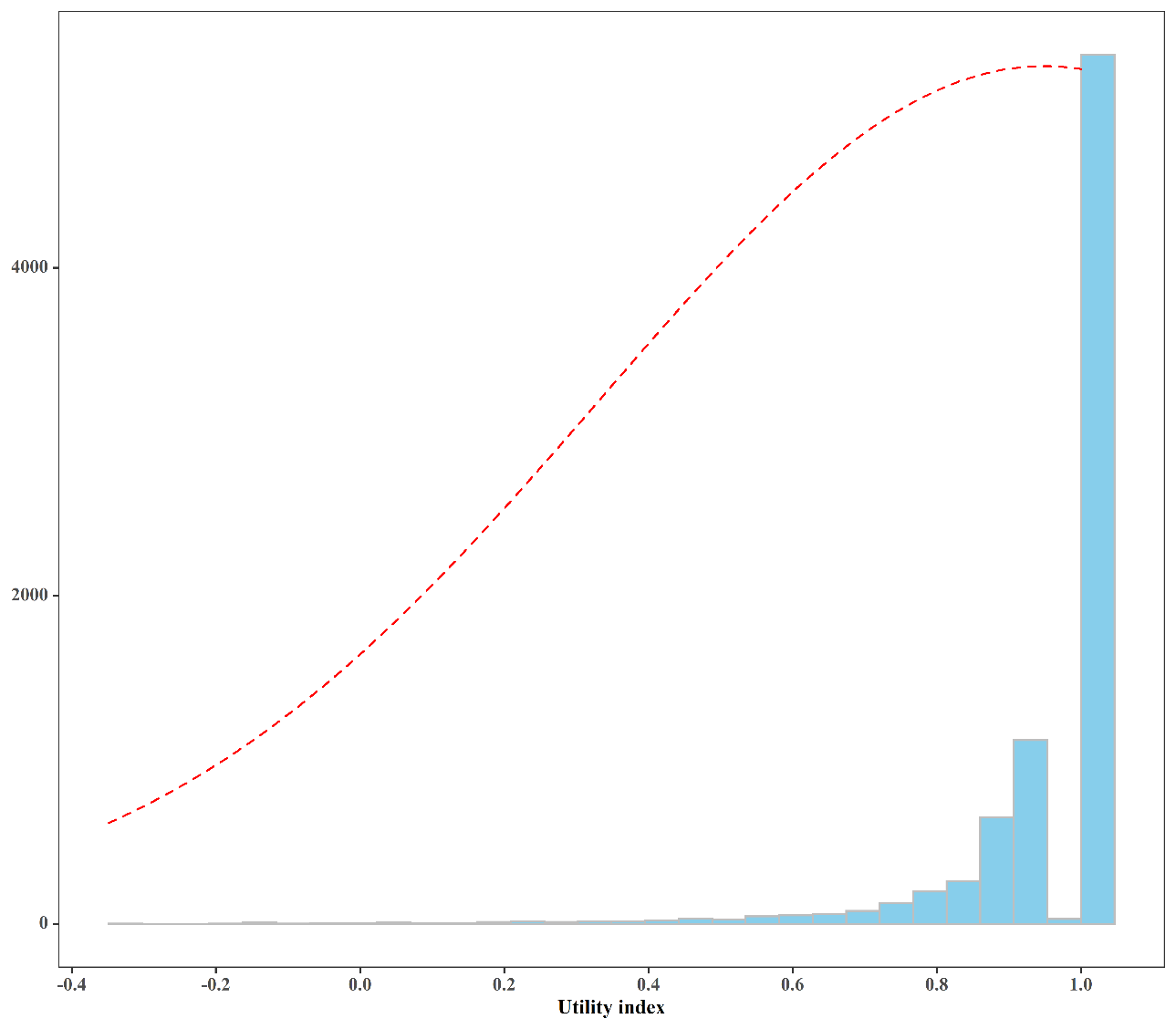


**Supplementary figure 1. The distribution of utility index of hypertensive patients.**

**Code**


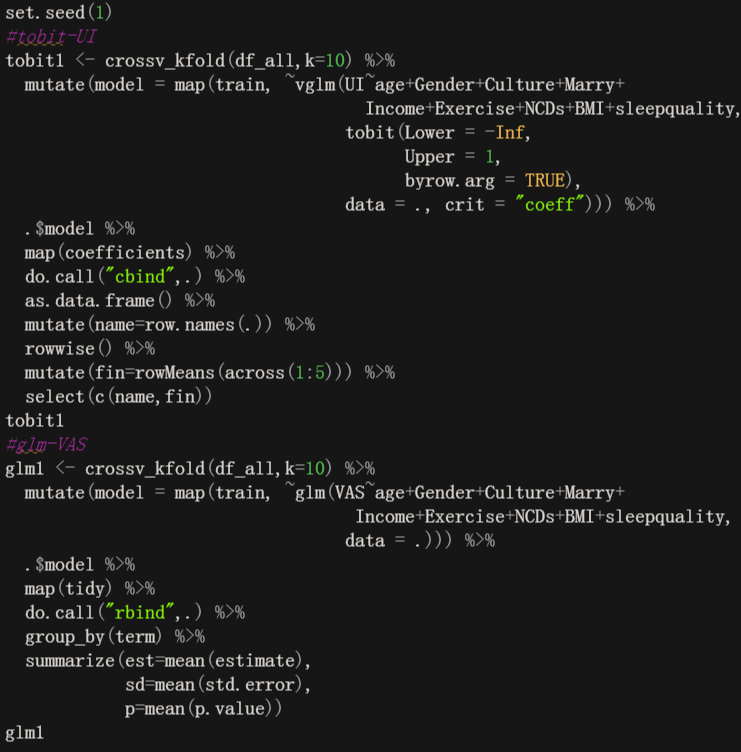

Supplement: Supplementary file 1 — Additional file 1. Supplementary appendix: Supplementary table 1. The association between hypertension and HRQoL. Supplementary table 2. Self-reported health problems of respondents. Supplementary table 3. Results of 10-folds cross-validation. Supplementary figure 1. The distribution of utility index of hypertensive patients. [file 12955_2021_1879_MOESM1_ESM.docx]
